# Postoperative red blood cell distribution width predicts functional outcome in aneurysmal subarachnoid hemorrhage after surgical clipping: A single-center retrospective study

**DOI:** 10.3389/fneur.2022.1036433

**Published:** 2022-12-22

**Authors:** Long Zhao, Yi Zhang, Ping Lin, Weida Li, Xingyuan Huang, Hangyang Li, Mingkai Xia, Xinlong Chen, Xi Zhu, Xiaoping Tang

**Affiliations:** ^1^Department of Neurosurgery, Affiliated Hospital of North Sichuan Medical College, Nanchong, China; ^2^School of Clinical Medicine, North Sichuan Medical College, Nanchong, China; ^3^Neurosurgical Research Center, Affiliated Hospital of North Sichuan Medical College, Nanchong, China; ^4^School of Psychiatry, North Sichuan Medical College, Nanchong, China; ^5^School of Medical Imaging, North Sichuan Medical College, Nanchong, China; ^6^Outpatient Department, Affiliated Hospital of North Sichuan Medical College, Nanchong, China

**Keywords:** aneurysmal subarachnoid hemorrhage, red blood cell distribution width, surgical clipping, functional outcome, predictor

## Abstract

**Objective:**

Red blood cell (RBC) parameters are associated with outcomes following aneurysmal subarachnoid hemorrhage (aSAH), but their predictive value remains uncertain. This study aimed to detect the association between RBC parameters and functional outcome in aSAH patients undergoing surgical clipping.

**Methods:**

This retrospective observational study included aSAH patients who underwent surgical clipping at Affiliated Hospital of North Sichuan Medical College between August 2016 and September 2019. The functional outcome following aSAH was assessed by modified Rankin Scale (mRS), and mRS 3–6 was defined as poor functional outcome.

**Results:**

Out of 187 aSAH patients included (62% female, 51–66 years old), 73 patients had poor functional outcome. Multivariate logistic regression of admission parameters showed that World Federation of Neurosurgical Societies (WFNS) grade (odds ratio [95% CI]: 1.322 [1.023–1.707], *p* = 0.033) and white blood cell (WBC) (odds ratio [95% CI]: 1.136 [1.044–1.236], *p* = 0.003) were independently associated with poor functional outcome. In postoperative parameters, RBC distribution width (RDW) (odds ratio [95% CI]: 1.411 [1.095–1.818], *p* = 0.008), mean platelet volume (MPV, odds ratio [95% CI]: 1.253 [1.012–1.552], *p* = 0.039) and admission WFNS grade (odds ratio [95% CI]: 1.439 [1.119–1.850], *p* = 0.005) were independently associated with poor functional outcome. The predictive model including WFNS grade, admission WBC, and postoperative RDW and MPV had significantly higher predictive power compared to WFNS grade alone (0.787 [0.722–0.852] vs. 0.707 [0.630–0.784], *p* = 0.024). The combination of WFNS grade and WBC on admission showed the highest positive predictive value (75.5%) and postoperative RDW and MPV combined with admission WFNS grade and WBC showed the highest negative predictive value (83.7%).

**Conclusion:**

Postoperative RDW is independently associated with poor functional outcome in aSAH patients undergoing surgical clipping. A combined model containing postoperative RDW may help predict good outcome in patients with aSAH after timely aneurysm clipping.

## 1. Introduction

Aneurysmal subarachnoid hemorrhage (aSAH), a severe type of hemorrhagic stroke, results from the rupture of intracranial aneurysms (1). With significant morbidity and mortality, such disease continues to be a clinical emergency despite advances in strategies of diagnosis, treatment, and neurocritical care (2). When a rupture occurs, guidelines recommend the repair be conducted as soon as possible (3, 4). Though endovascular coiling is widely performed, some forms of aSAH are suitable for surgical clipping. This requires complicated perioperative management and leads to clinical problems, such as the relationship between anemia, intraoperative blood loss and outcomes, which are still under investigation. Therefore, biomarkers predicting outcomes after surgical clipping are needed for advanced clinical care.

Red blood cell (RBC) parameters have been reported to predict poor outcomes after an aSAH event. Postoperative hemoglobin, an anemia biomarker, is observed to be an independent risk factor of poor neurological outcomes (5). Gong et al. found that mean corpuscular volume (MCV) and mean corpuscular hemoglobin (MCH) are predictors of cognitive impairment (6). Recent studies also have shown that RBC distribution width (RDW) is associated with outcome indicators such as functional outcome, mortality, delayed cerebral ischemia (DCI) and cerebral infarction following aSAH (7–9). Since the role of blood transfusion in the management of aSAH is still controversial and there is a lack of practical indicators for reference in the management of blood volume clinically, it is of great significance to continue evaluating the role of RBC parameters in aSAH.

Although several studies discussed the predictive value of RBC parameters, few considered the craniotomy performed in relation to the analysis, which may affect RBC parameters and outcome. Moreover, these studies have not been externally validated. It is unclear whether these results are broadly applicable and whether postoperative RBC parameters are associated with outcomes of aSAH patients after clipping. Therefore, this retrospective case-control study was conducted to comprehensively analyze the relationship between RBC parameters on admission and after surgical clipping and functional outcome of aSAH patients, and to screen parameters with independent predictive value.

## 2. Materials and methods

### 2.1. Study design and patients

This retrospective observational study included consecutive patients with aSAH admitted to Affiliated Hospital of North Sichuan Medical College between August 2016 and September 2019. The inclusion criteria were: (1) age >18 years; (2) patients with aSAH confirmed by computed tomographic angiography or digital subtraction angiography; (3) admitted within 24 h of initial symptom onset; (4) undergoing surgical clipping within 72 h of the onset; (5) complete blood count (CBC) test was completed within 24 h after admission and within 6 h after surgery. The exclusion criteria were: (1) patients with a history of primary or secondary central nervous system diseases, acute or chronic infections, or systemic diseases; (2) patients with trauma, surgery, bleeding events or blood donation within 3 months; (3) patients with incomplete information. This study was performed according to the TRIPOD statement (10), followed the revised Declaration of Helsinki and was approved by the ethics committee of Affiliated Hospital of North Sichuan Medical College. Patients' consent was waived due to the retrospective nature of this study.

### 2.2. Data collection and definition

Data were collected from the electronic medical records, including demographics (age, gender), addictions (smoking and alcohol consumption), medical history (hypertension, diabetes mellitus), admission clinical grades, aneurysm characteristics, and admission and postoperative CBC parameters. Clinical grades included the World Federation of Neurosurgical Society (WFNS) grade and modified Fisher (mFisher) grade. Aneurysm characteristics included location and number. Location was divided into “anterior” and “posterior” circulation, and number was divided into “single” and “multiple”. RBC parameters including hemoglobin, RBC, hematocrit, MCV, MCH, mean corpuscular hemoglobin concentration (MCHC), and RDW were collected on admission and postoperatively. Other CBC parameters reported as outcome predictors were also collected, including white blood cell (WBC), neutrophil, lymphocyte, monocyte, platelet and mean platelet volume (MPV) (11–13). Differences between admission and postoperative RBC parameters were calculated by subtracting the admission values from the postoperative values. For data accuracy and comparability, only the first CBC results after admission and surgery were collected separately.

Functional outcome was assessed through telephone interview or outpatient visit at 3 months by fixed staff who was blind to laboratory data of patients, following the modified Rankin scale (mRS) (14). The mRS is scored 0–6: 0 (no symptoms), 1 or 2 (functional independence), 3 (moderate handicap), 4 or 5 (moderate to severe handicap) and 6 (death). We defined good functional outcome as 3-month mRS between 0 and 2 and poor outcome as mRS between 3 and 6 (15). Patients were dichotomized into two groups (mRS 0–2 vs. mRS 3–6).

### 2.3. Statistical analysis

Statistical analysis was performed with SPSS Statistics 26 (IBM, Armonk, NY, USA), and *p* < 0.05 was considered significant. Categorical variables were presented as number (percentage) and compared by Chi-square or Fisher's exact test. Continuous variables that conformed to normal distribution were expressed as mean ± standard deviation and compared by Student's *t*-test. Continuous variables that conformed to skewed distribution were expressed as median with interquartile range (25th−75th percentile) and compared by the Mann-Whitney U test, which was also applied to rank variables. Multivariate logistic regression was performed to identify the independent predictors of poor functional outcome. Variables considered clinically relevant or with *p* < 0.05 in univariate analyses were incorporated into multivariate logistic regression models. Receiver operating characteristic (ROC) analysis was conducted to evaluate the predictive value and to determine the cutoff value by calculating Youden's index. Delong's test compared areas under ROC curves (AUCs).

## 3. Results

### 3.1. Baseline characteristics

**Figure 1:**
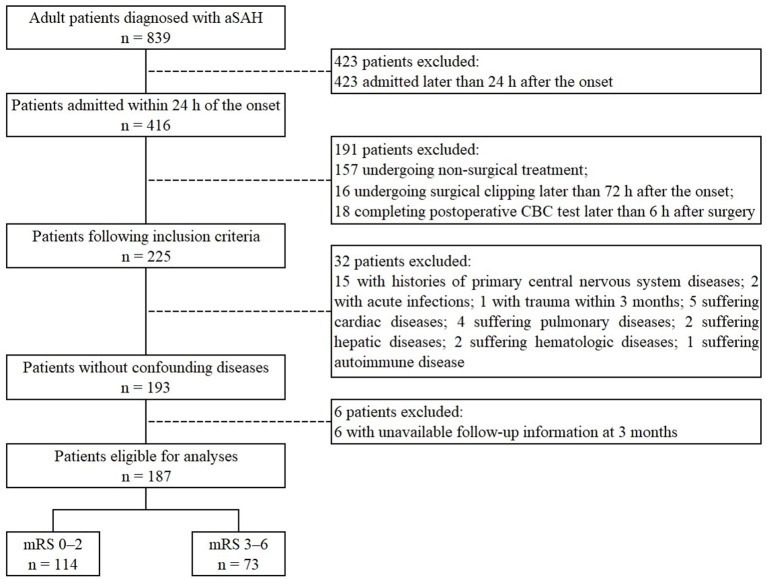
Flowchart of patients included in the study. aSAH, aneurysmal subarachnoid hemorrhage; CBC, complete blood count; mRS, modified Rankin Scale.

A total of 187 patients were included in this study, of which 114 (61.0%) had good functional outcome while 73 (39.0%) had poor functional outcome, including 15 patients (8.0%) who died within 3 months of the onset (Figure 1). Of the included cases, 122 were included in a published article to establish an early predictive nomogram for DCI after aSAH (13). Parameters of demographics, addictions, medical history and aneurysm characteristics showed no significant differences between two groups. In contrast, parameters of admission clinical grades differed significantly between two groups (WFNS grade, *p* < 0.001; mFisher grade, *p* = 0.011; Table 1).

**Table 1:** Univariate analysis of baseline characteristics and CBC parameters associated with poor functional outcome at 3 months.

**Characteristics**	**Total**	**mRS at 3 Months**		***p*-value**
	**(*n* = 187)**	**0–2 (*n* = 114)**	**3–6 (*n* = 73)**	
**Demographics**				
Female	116 (62.0%)	72 (63.2%)	44 (60.3%)	0.692
Age (year)	59 (51–66)	55 (50–67)	62 (53–66)	0.080
**Addictions**				
Smoking	35 (18.7%)	21 (18.4%)	14 (19.2%)	0.897
Alcohol consumption	28 (15.0%)	17 (14.9%)	11 (15.1%)	0.977
**Medical history**				
Hypertension	101 (54.0%)	59 (51.8%)	42 (57.5%)	0.439
Diabetes mellitus	4 (2.1%)	1 (0.9%)	3 (4.1%)	0.331
**Clinical grades**				
WFNS grade	2 (1–4)	1 (1–3)	3 (1–4)	**<0.001**
mFisher grade	3 (3–4)	3 (2–4)	3 (3–4)	**0.011**
**Aneurysm characteristics**				
Location				0.780
Anterior	179 (95.7%)	110 (96.5%)	69 (94.5%)	
Posterior	8 (4.3%)	4 (3.5%)	4 (5.5%)	
Number				0.984
Single	151 (80.7%)	92 (80.7%)	59 (80.8%)	
Multiple	36 (19.3%)	22 (19.3%)	14 (19.2%)	
**Admission CBC**				
WBC (×10^9^/L)	12.54 (9.64–15.74)	11.46 (8.90–14.00)	14.69 (11.81–17.90)	**<0.001**
Neutrophil (×10^9^/L)	11.32 (8.53–14.49)	10.30 (7.61–12.92)	13.18 (10.38–15.88)	**<0.001**
Lymphocyte (×10^9^/L)	0.81 (0.59–1.16)	0.81 (0.58–1.20)	0.81 (0.62–1.11)	0.812
Monocyte (×10^9^/L)	0.42 (0.28–0.65)	0.37 (0.23–0.51)	0.60 (0.36–0.84)	**<0.001**
Hemoglobin (g/L)	128 ± 17	130 ± 14	125 ± 19	0.069
RBC (10^12^/L)	4.30 (3.89–4.57)	4.31 (3.96–4.56)	4.30 (3.83–4.59)	0.546
Hematocrit	0.389 (0.358–0.423)	0.392 (0.364–0.423)	0.383 (0.348–0.423)	0.210
MCV (fL)	92.0 (89.8–96.0)	92.0 (89.9–96.1)	93.1 (89.8–95.5)	0.803
MCH (pg)	30.2 (29.1–31.4)	30.5 (29.3–31.5)	30.0 (28.8–31.1)	0.103
MCHC (g/L)	326 (320–333)	328 (321–334)	324 (318–331)	**0.011**
RDW (%)	13.2 (12.6–13.7)	13.1 (12.5–13.6)	13.4 (12.8–14.2)	**<0.001**
Platelet (×10^9^/L)	186.8 ± 51.6	186.2 ± 46.7	187.8 ± 58.7	0.841
MPV (fL)	11.5 ± 1.7	11.3 ± 1.8	11.9 ± 1.6	**0.039**
**Postoperative CBC**				
WBC (×10^9^/L)	11.72 (9.43–14.77)	10.82 (9.01–13.80)	12.93 (10.29–16.86)	**0.001**
Neutrophil (×10^9^/L)	9.80 (7.79–13.01)	8.89 (7.46–11.73)	11.77 (8.56–14.72)	**<0.001**
Lymphocyte (×10^9^/L)	0.85 (0.59–1.23)	0.88 (0.59–1.32)	0.85 (0.59–1.15)	0.655
Monocyte (×10^9^/L)	0.63 (0.45–0.95)	0.59 (0.41–0.88)	0.69 (0.51–1.04)	**0.041**
Hemoglobin (g/L)	110 (99–118)	111 (101–119)	108 (96–115)	0.081
RBC (10^12^/L)	3.66 ± 0.52	3.66 ± 0.44	3.66 ± 0.63	0.941
Hematocrit	0.339 (0.310–0.367)	0.340 (0.314–0.367)	0.335 (0.299–0.367)	0.261
MCV (fL)	93.6 (90.6–96.9)	93.7 (91.1–96.8)	92.6 (90.1–96.9)	0.254
MCH (pg)	30.3 (29.2–31.1)	30.3 (29.3–31.3)	30.0 (29.0–30.8)	0.097
MCHC (g/L)	322 (315–328)	324 (316–328)	321 (314–328)	0.116
RDW (%)	13.3 (12.8–14.1)	13.1 (12.6–13.6)	13.9 (13.2–14.5)	**<0.001**
Platelet (×10^9^/L)	144 (115–172)	143 (118–168)	144 (112–177)	0.872
MPV (fL)	11.6 ± 1.6	11.3 ± 1.6	12.0 ± 1.5	**0.004**

Boldface type represents statistical significance.

Values are n (%), mean ± standard deviation or median (25−75%).

CBC, complete blood count; mRS, modified Rankin Scale; WFNS, World Federation of Neurosurgical Societies; mFisher, modified fisher; WBC, white blood cell; RBC, red blood cell; MCV, mean corpuscular volume; MCH, mean corpuscular hemoglobin; MCHC, mean corpuscular hemoglobin concentration; RDW, red blood cell distribution width; MPV, mean platelet volume.

### 3.2. Admission CBC parameters

Admission CBC parameters showed that WBC (11.46 [8.90–14.00] vs. 14.69 [11.81–17.90], *p* < 0.001), neutrophil (10.30 [7.61–12.92] vs. 13.18 [10.38–15.88], *p* < 0.001), monocyte (0.37 [0.23–0.51] vs. 0.60 [0.36–0.84], *p* < 0.001) and RDW (13.1 [12.5–13.6] vs. 13.4 [12.8–14.2], *p* < 0.001) in patients with poor functional outcome were significantly higher than those in patients with good functional outcome (Table 1). Patients with poor functional outcome also had significantly lower MCHC (328 [321–334] vs. 324 [318–331], *p* = 0.011; Table 1). Multivariate logistic regression showed that WFNS grade (odds ratio [95% CI]: 1.322 [1.023–1.707], *p* = 0.033) and WBC (odds ratio [95% CI]: 1.136 [1.044–1.236], *p* = 0.003) predicted poor functional outcome independently (Table 2).

**Table 2:** Multivariate logistic regression of baseline characteristics and admission CBC parameters associated with poor functional outcome at 3 months.

**Characteristics**	**OR**	**95% CI**	***p-*value**
WFNS grade	1.322	1.023–1.707	**0.033**
mFisher grade	1.165	0.796–1.706	0.432
WBC	1.136	1.044–1.236	**0.003**
MCHC	0.975	0.939–1.013	0.196
RDW	1.327	0.918–1.919	0.132
MPV	1.199	0.982–1.462	0.074

Boldface type represents statistical significance.

CBC, complete blood count; OR, odds ratio; CI, confidence interval; WFNS, World Federation of Neurosurgical Societies; mFisher, modified fisher; WBC, white blood cell; MCHC, mean corpuscular hemoglobin concentration; RDW, red blood cell distribution width; MPV, mean platelet volume.

### 3.3. Postoperative CBC parameters

In postoperative CBC parameters, WBC (10.82 [9.01–13.80] vs. 12.93 [10.29–16.86], *p* = 0.001), neutrophil (8.89 [7.46–11.73] vs. 11.77 [8.56–14.72], *p* < 0.001), monocyte (0.59 [0.41–0.88] vs. 0.69 [0.51–1.04], *p* = 0.041), RDW (13.1 [12.6–13.6] vs. 13.9 [13.1–14.5], *p* < 0.001) and MPV (11.3 ± 1.6 vs. 12.0 ± 1.5, *p* = 0.004) in patients with poor functional outcome were significantly higher than those with good functional outcome (Table 1). Results of multivariate logistic regression revealed that WFNS grade (odds ratio [95% CI]: 1.439 [1.119–1.850], *p* = 0.005), MPV (odds ratio [95% CI]: 1.253 [1.012–1.552], *p* = 0.039) and RDW (odds ratio [95% CI]: 1.411 [1.095–1.818], *p* = 0.008) independently predicted poor functional outcome (Table 3).

**Table 3:** Multivariate logistic regression of baseline characteristics and postoperative CBC parameters associated with poor functional outcome at 3 months.

**Characteristics**	**OR**	**95% CI**	***p*-value**
WFNS grade	1.439	1.119–1.850	**0.005**
mFisher grade	1.189	0.824–1.718	0.355
WBC	1.083	0.993–1.182	0.073
RDW	1.411	1.095–1.818	**0.008**
MPV	1.253	1.012–1.552	**0.039**

Boldface type represents statistical significance.

CBC, complete blood count; OR, odds ratio; CI, confidence interval; WFNS, World Federation of Neurosurgical Societies; mFisher, modified fisher; WBC, white blood cell; RDW, red blood cell distribution width; MPV, mean platelet volume.

### 3.4. Differences between admission and postoperative RBC parameters

The variation of RBC parameters was analyzed and only RDW showed significant difference. The difference of RDW in patients with poor functional outcome was higher than those with good functional outcome (0.2 [−0.2 to 1.0] vs. 0.0 [−0.3 to 0.4], *p* = 0.010).

### 3.5. ROC analysis

**Figure 2:**
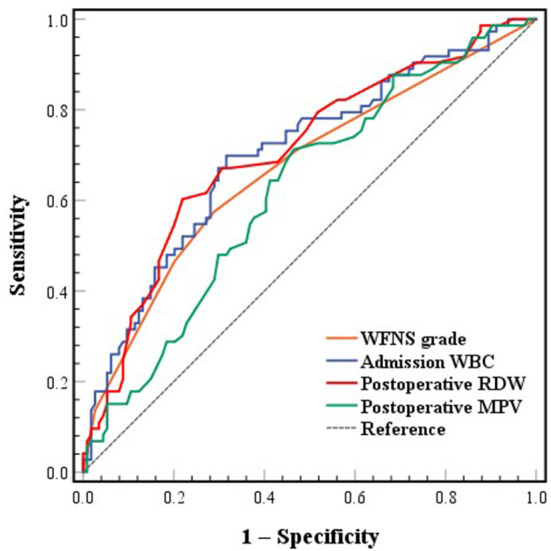
ROC curves of WFNS grade, admission WBC, postoperative RDW and MPV associated with poor functional outcome at 3 months. ROC, receiver operating characteristic; WFNS, World Federation of Neurosurgical Societies; WBC, white blood cell; RDW, red blood cell distribution width; MPV, mean platelet volume.

The AUCs of WFNS grade, admission WBC and postoperative RDW and MPV were 0.670 (0.589–0.750), 0.704 (0.626–0.781), 0.707 (0.630–0.784) and 0.623 (0.542–0.704), respectively (Figure 2). The best cutoff point of WFNS grade predicting poor functional outcome (Youden's index = 0.286) was 2.5, where the sensitivity was 57.5% and the specificity was 71.1%. The positive predictive value (PPV) was 56.0% and the negative predictive value (NPV) was 72.3%. The best cutoff point of admission WBC predicting poor functional outcome (Youden's index = 0.383) was 12.875×10^9^/L, where the sensitivity was 69.9% and the specificity was 68.4%. The PPV was 58.6% and the NPV was 78.0%. The best cutoff point of postoperative RDW predicting poor functional outcome (Youden's index = 0.383) was 13.65%, where the sensitivity was 60.3% and the specificity was 78.1%. The PPV was 63.8% and the NPV was 75.4%. The best cutoff point of postoperative MPV predicting poor functional outcome (Youden's index = 0.247) was 11.25 fL, where the sensitivity was 71.2% and the specificity was 53.5%. The PPV was 49.5% and the NPV was 74.4%. There was no significant difference between the AUCs of the four ROC curves (WFNS grade vs. admission WBC, *p* = 0.462; WFNS grade vs. postoperative RDW, *p* = 0.477; WFNS grade vs. postoperative MPV, *p* = 0.419; admission WBC vs. postoperative RDW, *p* = 0.949; admission WBC vs. postoperative MPV, *p* = 0.182; postoperative RDW vs. postoperative MPV, *p* = 0.112). The AUC of postoperative RDW was significantly larger than the difference of RDW (*p* = 0.020).

**Figure 3:**
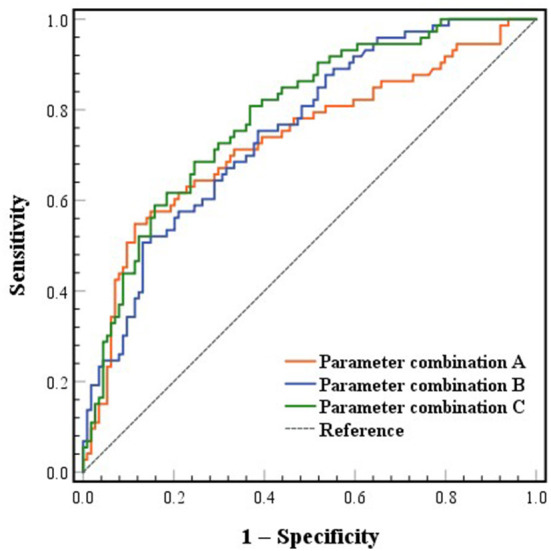
ROC curves of combined parameters predicting poor functional outcome at 3 months. ROC, receiver operating characteristic. Parameter combination A included World Federation of Neurosurgical Societies (WFNS) grade and admission white blood cell (WBC). Parameter combination B included WFNS grade and postoperative red blood cell distribution width (RDW) and mean platelet volume (MPV). Parameter combination C included WFNS grade, admission WBC and postoperative RDW and MPV.

Three combined diagnostic models based on WFNS grade, admission WBC and postoperative RDW and MPV were established and evaluated to achieve better predictive value. The AUC of parameter combination A (WFNS grade and admission WBC) and B (WFNS grade and postoperative RDW and MPV) was 0.734 (0.657–0.811) and 0.753 (0.685–0.822), respectively (Figure 3). The sensitivity was 54.8% and the specificity was 88.6% at the best cutoff point of parameter combination A (Youden's index = 0.434). The PPV was 75.5% and the NPV was 75.4%. The sensitivity was 50.7% and the specificity was 86.8% at the best cutoff point of parameter combination B (Youden's index = 0.375). The PPV was 71.2% and the NPV was 73.3%. The AUC of parameter combination C (all four parameters) was 0.787 (0.722–0.852) (Figure 3). The sensitivity was 80.8% and the specificity was 63.2% at the best cutoff point of parameter combination C (Youden's index = 0.440). The PPV was 58.4% and the NPV was 83.7%. The AUC of parameter combination C was higher than parameter combination A and B with statistical significance (parameter combination A vs. parameter combination C, *p* = 0.029; parameter combination B vs. parameter combination C, *p* = 0.048). The AUC of parameter combination A was significantly higher than WFNS grade (*p* = 0.018). The AUC of parameter combination B was significantly higher than WFNS grade and postoperative MPV (parameter combination B vs. WFNS grade, *p* = 0.005; parameter combination B vs. postoperative MPV, *p* = 0.001). The AUC of parameter combination C was higher than all four parameters alone (parameter combination C vs. WFNS grade, *p* < 0.001; parameter combination C vs. admission WBC, *p* = 0.009; parameter combination C vs. postoperative RDW, *p* = 0.024; parameter combination C vs. postoperative MPV, *p* < 0.001).

## 4. Discussion

This study showed that admission WBC and postoperative RDW and MPV were associated with poor functional outcome in aSAH patients undergoing surgical clipping, and combining them with WFNS grade could improve the predictive value. It also revealed that combining RBC parameters could substantially increase the clinical prediction of functional outcome.

RDW changes dynamically with the development of aSAH. When an aSAH happens, body damage is inevitable. Clinicians and scientists have been investigated pathophysiological mechanisms following aSAH for decades. Several studies have found that the rupture of an aneurysm leads to a series of mechanisms such as cerebral edema, inflammation, oxidative stress and microthrombosis (16–18). A recent study has shown that the toll-like receptor (TLR) 4/myeloid differentiation primary response protein (MyD) 88/nuclear factor (NF)-κB pathway plays an essential role in the neuroinflammation following aSAH (19). After the activation of TLR4 by endogenous ligands with damage-associated molecular patterns produced after aSAH, the MyD88-dependent pathway activates the transcription factor NF-κB. It also produces pro-inflammatory cytokines such as tumor necrosis factor (TNF)-α, interleukins, intercellular adhesion molecule-1 (19), etc. According to the research by Wang et al. (20), RDW predicts significant inflammation in patients with autoimmune hepatitis. RDW is also associated with serum TLR4 and TNF-α (21, 22), both belonging to the TLR4/MyD88/NF-κB pathway. Therefore, it could be inferred that RDW is a biomarker of inflammation following aSAH. In an animal experiment by Zhao et al. (21), RDW is associated with serum superoxide dismutase and malondialdehyde, representing oxidative stress. Oxidative stress reduces the survival rate of RBCs, causing immature RBCs to increase in circulation, contributing to an increase in RDW. Furthermore, RDW is associated with stroke, cerebral infarction and cerebral vein thrombosis (23, 24). The release of internal organelles and the externalization of vesicular contents in the maturation process of the immature RBCs may lead to the activation of the coagulation system and thrombosis (25). Studies mentioned above suggest that inflammatory response, oxidative stress and microthrombosis are all related to RDW changes after aSAH. Although the admission RDW in the group of poor outcome was found to be significantly higher than the group of good outcome, admission RDW did not predict functional outcome independently in this study. In contrast, WFNS grade and admission WBC, which were deemed to be confounders, showed statistical significance in multivariate logistic regression. Additionally, RDW may not change in such a short amount of time, and factors like nutritional status can also affect the level of RDW, which could explain the results of multivariate logistic regression.

In this study, postoperative RDW was found to independently predict poor functional outcome at 3 months after surgical clipping in patients with aSAH, whereas admission RDW was not a predictor. The elevation of RDW on admission may reflect pathophysiological damage mediated by bleeding, as it is generally known that RDW increases with blood loss. Since blood loss and occasional RBC transfusions are inevitable in craniotomy, an increase in RDW is likely to occur (26). Besides, inflammation, oxidative stress and a hypercoagulable state of blood are everyday events after craniotomy, all probably elevating the RDW level. Since these mechanisms also occur at the initial stage of aSAH, we infer that the disadvantages of surgical clipping may magnify the pathophysiological changes present on admission.

RDW has been widely used for the differential diagnosis of anemia over the past few decades (27, 28). Recent studies have shown that the function of RDW is mainly applied in cardiovascular and cerebrovascular diseases (29). In diagnosing acute myocardial infarction (AMI) among patients with chest pain and the discrimination of stroke subtypes in young patients, RDW demonstrates excellent diagnostic accuracy (30, 31). Other studies have suggested that RDW could also be a risk and outcome predictor. In patients with coronary artery diseases, RDW is associated with myocardial scar burden, impaired left ventricular function and postoperative cognitive dysfunction (32, 33). In patients suffering from atrial fibrillation, RDW enhances the risk of major adverse cardiovascular events, thromboembolic events and all-cause mortality (34–36). In patients undergoing ischemic stroke, RDW predicts worse neurological improvement and mortality (37, 38). RDW also shows predictive performance in hemorrhagic cerebrovascular diseases like spontaneous intracerebral hemorrhage (39) and aSAH (7–9), the latter of which our study focused on.

This study presents the first published analysis investigating the relationship between postoperative RDW and functional outcome in aSAH patients undergoing surgical clipping. Three previous studies performed dynamic monitoring of RDW. Fontana et al. monitored RDW daily from the day of admission for a maximum of 7 days during the intensive unit stay (7), and Siegler et al. (9) and Chugh et al. (15) collected RDW data continuously for up to 10 days or longer after aSAH onset. However, these studies did not mention whether patients received surgical treatment and when the surgical treatment was performed. As we explained above, admission and postoperative RDW are different, so evaluating RDW levels by calculating the maximum or mean throughout the aSAH course is likely to cause bias. In addition, high RDW levels at different periods will correspond to different strategies of clinical management. Hence, the separate assessment of admission and postoperative RDW may provide more practical guidance for the perioperative management of aSAH. Therefore, we selected patients who only underwent surgical clipping to reduce the confounding bias.

In addition to screening for independent predictors of poor functional outcome, we evaluated the differentiation and predictive potential of each predictor individually and in combination for functional outcome by comparing AUCs and calculating PPVs and NPVs. The result of AUC comparison showed that combined models had a higher degree of differentiation than single predictors and the combined model of all four predictors was the highest. The PPVs indicated that the combination of WFNS grade and admission WBC appeared to provide the best predictive power of poor outcome. However, the combination of all parameters had the highest NPV, 83.7%, which could be used to predict good outcome. These results suggest that adding RDW to the predictive model improves the predictive power and has greater potential for predicting good outcome.

The RDW test is an excellent strategy of biomarker detection because it is easy to implement, inexpensive, and noninvasive. Since RDW is independently associated with functional outcome, it could guide clinical decisions in the perioperative management of aSAH, especially in the postoperative management. If the application of RDW is combined with demographics, medical history, clinical grades, radiological data and other laboratory data, it may contribute to better assessment, improve functional outcome and bring great benefit to patients. For example, RBC transfusion is usually given at the discretion of treating physicians when patients suffer blood loss in craniotomy or (and) anemia caused by other factors (40). However, the effect of RBC transfusion in aSAH can be detrimental. Several studies have shown that RBC transfusion is associated with unfavorable functional outcome indicators, such as poor functional outcome, mortality, acute lung injury, acute respiratory distress syndrome and thrombotic events (41–43). RDW is elevated during the regeneration of RBCs after blood loss and help diagnose some types of anemia. However, there is no specific guideline for RBC transfusion in aSAH. Thus, we hypothesize that combining hemoglobin and RDW to adjust transfusion practice may contribute to better functional outcome.

## 5. Limitations

This study has several limitations. First, it is a retrospective study performed at a single center with a small sample size, which is less persuasive than a large prospective study. Second, although baseline information regarding demographics, addictions, medical history, admission clinical grades and aneurysm characteristics was collected, some data like nutritional status, reticulocyte count and RBC transfusion records were not obtained. Third, other outcome indicators such as mortality, cerebral infarction, cognitive impairment, etc., were not collected. Finally, dynamic monitoring of RDW wasn't performed, which could provide more in-depth analysis.

## 6. Conclusion

Postoperative RDW is independently associated with poor functional outcome in aSAH patients undergoing surgical clipping. Combing postoperative RDW and MPV with WFNS grade and WBC on admission may help predict good outcome more accurately. Further research is demanded to determine the clinical value and relevant mechanisms.

## Data availability statement

The raw data supporting the conclusions of this article will be made available by the authors, without undue reservation.

## Ethics statement

The studies involving human participants were reviewed and approved by Medical Ethics Committee of Affiliated Hospital of North Sichuan Medical College. The Ethics Committee waived the requirement of written informed consent for participation.

## Author contributions

This study was conceptualized and designed by LZ, YZ, XZ, and XT. Data collection and analysis were performed by LZ, YZ, PL, WL, XH, HL, MX, and XC. The original manuscript was written by LZ and YZ. Review and revision of the manuscript were performed by XZ and XT. All authors read and approved the final manuscript. All authors commented on previous versions of the manuscript.
